# Editorial: Accelerated Globalization and Its Impact to the World's Health Care Achievement

**DOI:** 10.3389/fpubh.2021.690239

**Published:** 2021-06-07

**Authors:** Mihajlo Jakovljevic, Márta Péntek, Tissa Wijeratne, Guvenc Kockaya, Louis-François Pau

**Affiliations:** ^1^Institute of Comparative Economic Studies, Hosei University, Tokyo, Japan; ^2^Department of Global Health Economics and Policy, University of Kragujevac, Kragujevac, Serbia; ^3^HECON — Health Economics Research Center, Óbuda University, Budapest, Hungary; ^4^Department of Neurology and Stroke Services, Melbourne Medical School, Faculty of Medicine, Dentistry and Health Sciences, Western Health, University of Melbourne, Parkville, VIC, Australia; ^5^ECONiX Research, Analysis and Consultancy Plc., Istanbul, Turkey; ^6^Copenhagen Business School, Frederiksberg, Denmark; ^7^Rotterdam School of Management (RSM), Netherlands Erasmus University, Rotterdam, Netherlands; ^8^Upgötva AB, Stockholm, Sweden

**Keywords:** globalization, health economics, health expenditure, spending, health financing, costs, health care, emerging markets

The traditional world economic order was inherited from the Colonial Age, with an assumed hierarchy of rich industrialized nations from the Northern hemisphere and largely poorer and underdeveloped nations in the Southern hemisphere. Capital investment and technology dissemination has historically moved southwards. Production of quality goods and services has occurred in the Global South using local affordable and skilled labor, while the same goods and services were mostly consumed by rich customers coming from the Global North or exported northwards to these same regions. Over the course of several consecutive industrial revolutions this mainstream flow remained prominent and visible throughout most of the XVIII–XX centuries (1).

This traditional pattern met a serious challenge during the post-WWII decades. The Cold War lasted for 45 years and brought heavy industrial development among the so-called Second World socialist economies led by the USSR. Many of the cutting-edge technologies of the time were disseminated by state-sponsored talent breeding programs in communist countries with the space and military industries being the most prominent examples (2). In 1989–1991 the era of the Cold War ended, and there was an acceleration of globalization worldwide driven to a large extent by commercial interests of powerful multinational businesses, but with very little dissemination of health-related technologies. Among the consequences of this change were market-driven dissemination of industrial technologies toward many of the historically under-developed economies, combined with an outsourcing of manufacturing to these areas. A milestone example of this profound change was the economic reforms introduced by Deng Xiaoping in the People's Republic of China in 1978, which has resulted in China following an exceptionally upward economic pathway since 1989 (3).

Changes taking place over the next 30 years shall gradually become known as the Rise of Emerging Markets led by the BRIC nations (Brazil, Russia, India, China) (4). Few other LMICs nations such as Mexico, Nigeria, South Africa, Singapore, Indonesia, Vietnam remain characterized by long term rapid real GDP growth but at significantly smaller scale of national economies (5). Happening alongside an economic slowdown in Western economies, particularly since the last global recession of 2007–2017, this evolution has changed the entire global macroeconomic landscape and reflects heavily in the health care arena. A recently released Brookings Institute report based on International Monetary Fund (IMF) data provides a clear illustration of this change. The report observes that EM7 nations (seven major emerging markets: BRIC + Turkey, Indonesia, and Mexico) have contributed one-half of the world's economic growth (in terms of real GDP growth rates) in comparison to G7 countries (USA, Japan, Germany, UK, France, Canada, Italy), which have contributed jointly to only one-quarter of the world's growth in 2018–2019 (6). To emphasize this point further, at a previous Davos forum (7) it was stated that in the past decade China, as the classical case of an economic overachiever, contributed alone to approximately one-third of all global economic growth. This shift means that now we have an acceleration of South-South global trade. Major investors in the most rapidly developing world regions do not typically come from major Western, Organization for Economic Co-operation and Development (OECD) member economies, or Japan anymore (8). Instead, such capital and technology investments frequently are outsourced from China, India, Russia, or Middle Eastern countries. It is noteworthy to highlight that these South-South trade-flows include pharmaceutical ingredients and products incl. their manufacturing (India is an example of the global powerhouse in generics—copycat medicines manufacturing), affordable medical technologies, health-related tourism, but not yet health services. Huge chunk of this demand for health services is outsourcing from a massive growth of Non-communicable Diseases related morbidity across the Global South and LMICs nations (9). In the sense of these upcoming challenges and long term shortage of disposable resources to be allocated, cost-effective health care has become essential (10). Ongoing pandemics has essentially made these capacities overstretched even more (11).

This Research Topic was created in order to address the core challenges created by globalization for national health and socioeconomic systems worldwide (12). The ongoing 4.0 Industrial Revolution, robotics, and internet addiction issues are among its prominent consequences (13). Throughout its life cycle it has attracted a total of four contributions.

First one of them entitled: “Bootstrap ARDL on Health Expenditure, CO_2_ Emissions, and GDP Growth Relationship for 18 OECD Countries” has brought a surprising perspective on the dynamic relationship among CO_2_ emissions, health care expenditure, and GDP growth for the 18 OECD countries over the period of 1975–2017. In their conclusive remarks authors observe that limitations of national CO_2_ emissions remain the mainstay of OECD countries policies. Recommendations were to adopt measures and policies to protect the quality of the environment to reduce the occurrence of health diseases (Wang et al.).

Another contribution entitled: “Realization of the EU's Cohesion Policy in Health Care in the Visegrad Group Countries in the Perspective 2014–2020.” An overview of European Union's cohesion policy in the field of health care was provided for Poland, the Czech Republic, the Slovak Republic and Hungary—the Visegrad Group–VG4—in the period of 2014–2020. The VG4 countries appears to have distinctive benefits from these EU programmes and managed to adjust their use to their internal circumstances and needs (Holecki, Kowalska-Bobko et al.).

The third piece published in this Topic was: “Global Elderly Migrations and Their Impact on Health Care Systems.” Healthcare System Indices were used to assess the global flows of migrations of elderly citizens. It appears that seniors make a decision about migration based on a combination of factors such as the quality and availability of medical services and the simultaneous low or average cost of living. The effective medical tourism and long term movement of senior citizens frequently suffering from multiple NCDs brings up the systemic challenge for the accepting societies (Holecki, Rogalska et al.).

The last article submitted was: “A Polish Pilot Programme of Coordinated Care: A Herald of Change or a Missed Opportunity? A Critical Debate.” Back on 1 July 2018 the Polish government launched POZ Plus—a pilot programme of coordinated primary care. As per authors' judgement, if Polish PHC is about to gain more prominence—POZ Plus has to be implemented on a much wider scale. The hottest three policy bottlenecks to be addressed are healthcare funding, workforce strategy, and collaboration between the government, and other stakeholders (Karasiewicz et al.).

Globalization has also occurred through technologies, and few have been as deep as the Internet. Besides benefits and opportunities, it has also brought direct and indirect negative effects in the global health care field. Whereas, select mostly psychiatric problematic uses of the Internet (PUI) have been categorized by WHO, only very scant attention has been given e.g., to optometric and nutrition implications, and to behavioral changes linked to high global usage of mobile social networks (14). It is only recently that intense usage of telework linked to globalization has been shown to hit mental health limits. Health, content, and workplace regulations aimed at preserving global well-being spread in emerging markets conscious of national cultures, but not in unregulated developed areas. At stake is the need for research aimed at balancing off health risks and costs from excessive Internet usage, with information dissemination (15, 16).

## Author Contributions

MJ has prepared the manuscript draft while MP, TW, GK, and LFP have revised it for important intellectual content. All authors contributed to the article and approved the submitted version.

## Conflict of Interest

L-FP was an Independent Board Member for company Upgötva AB, Stockholm, Sweden. GK was partner and CEO of the company ECONiX Research, Analysis and Consultancy Plc., Istanbul, Turkey. The remaining authors declare that the research was conducted in the absence of any commercial or financial relationships that could be construed as a potential conflict of interest.
